# Paternal obesity and its transgenerational effects on gastrointestinal function in male rat offspring

**DOI:** 10.1590/1414-431X2020e11116

**Published:** 2021-05-31

**Authors:** M.P.R. Machado, L.A. Gama, A.P.S. Beckmann, A.T. Hauschildt, D.J.R. Dall'Agnol, J.R.A. Miranda, L.A. Corá, M.F. Américo

**Affiliations:** 1 Instituto de Biociências, Universidade Estadual Paulista Júlio de Mesquita Filho, Botucatu, SP, Brasil; 2 Instituto de Ciências Biológicas e da Saúde, Universidade Federal de Mato Grosso, Barra do Garças, MT, Brasil; 3 Departamento de Fisiologia e Farmacologia, Universidade Federal do Ceará, Fortaleza, CE, Brasil; 4 Faculdade de Ciências Agrárias, Biológicas, Engenharia e da Saúde, Universidade do Estado de Mato Grosso, Tangará da Serra, MT, Brasil; 5 Núcleo de Ciências Biológicas, NUCIB, Universidade Estadual de Ciências da Saúde de Alagoas (UNCISAL), Maceió, AL, Brasil

**Keywords:** Epigenetics, Gastric emptying, Intestinal transit, Monosodium glutamate

## Abstract

The interplay between obesity and gastrointestinal (GI) motility is contradictory, and the transgenerational influence on this parameter is unknown. We aimed to evaluate the GI function in a model of paternal obesity and two subsequent generations of their male offspring. Newborn male rats were treated with monosodium glutamate (MSG) and composed the F1 generation, while control rats (CONT) received saline. At 90 days, male F1 were mated with non-obese females to obtain male offspring (F2), which later mated with non-obese females for obtaining male offspring of F3 generation. Lee Index analysis was adopted to set up the obesity groups. Alternating current biosusceptometry (ACB) technique was employed to calculate GI transit parameters: mean gastric emptying time (MGET), mean cecum arrival time (MCAT), mean small intestinal transit time (MSITT), and gastric frequency and amplitude of contractions. Glucose, insulin, and leptin levels and duodenal morphometry were measured. F1 obese rats showed a decrease in the frequency and amplitude of gastric contractions, while obese rats from the F2 generation showed accelerated MGET and delayed MCAT and MSITT. Glucose and leptin levels were increased in F1 and F2 generations. Insulin levels decreased in F1, F2, and F3 generations. Duodenal morphometry was altered in all three generations. Obesity may have paternal transgenerational transmission, and it provoked disturbances in the gastrointestinal function of three generations.

## Introduction

Obesity is a multifactorial disease and has become a global pandemic. Over 650 million obese people (1,2) face a complex interplay between biological, epigenetic, psychosocial, environmental, and industrial factors (2,3).

Both non-genetic effects and genetic inheritance as well as epigenetic dysregulation can contribute to the predisposition to obesity (4). In this context, parental health or exposures can affect the lifetime health outcomes of offspring (4,5). Since maternal effects have been the focus of the transgenerational transmission of metabolic disorders (5–9), the role of paternal obesity in offspring metabolic programming has been investigated but still not fully elucidated (8,10).

Experimental models might contribute towards understanding the pathophysiological mechanisms and consequences concerning transgenerational obesity (6,9). When administered to neonatal rats, monosodium glutamate (MSG) is recognized to cause obesity and clinical manifestations of metabolic syndrome (6,11). MSG provokes hypothalamic lesions resulting in several endocrine disturbances, stunted growth, and obesity (7,12). MSG-induced maternal obesity results in spontaneously obese animals in the second generation (6).

Obesity is often associated with complications that affect cardiovascular, endocrine, and gastrointestinal (GI) systems (2,13,14). GI motility, in particular, plays a very critical role in the digestion and absorption of nutrients (15), and also participates in the control of appetite and satiety (13). However, the relationship between obesity and GI motility is conflicting, and transgenerational effects have not been evaluated properly (10,16). We hypothesized that obesity can alter parenteral GI function and such effect can also manifest in the male offspring. The aim of this study was to evaluate the GI function in a model of paternal obesity and in two subsequent generations of their male offspring.

## Material and Methods

### Experimental protocol

All experiment procedures were approved by the Ethics Committee on Animal Research of the Federal University of Mato Grosso (protocol number 23108.705702/13-9), following the 3Rs principle (Replacement, Reduction, and Refinement) of animal research. Wistar rats had free access to water and standard diet (Labina Presence®, USA) and were kept in individual cages under controlled conditions of temperature (22±1°C), humidity (50±10%), and a 12-h light/dark cycle.

For obesity induction, male neonatal Wistar rats received 4 mg/g body weight subcutaneous (sc) injections of MSG (Sigma-Aldrich, USA) on days 2, 4, 6, 8, and 10 of postnatal life. Control Wistar rats received subcutaneous injections of saline solution (0.9% NaCl) on the same days (6,9). Both groups were weaned at 21 days of age and maintained in controlled conditions.

The Lee index was applied to all the male rats at 90 days of life. This index is defined as the cube root of body weight (g) × 10 / naso-anal length (cm) and values >0.300 are classified as obese (6,9). All obese animals included in the MSG group were defined as F1 generation.

Obese rats of F1 generation were mated with non-obese adult female rats (90 days of age) and upon pregnancy detection (sperm-positive confirmation), pregnant rats were housed in individual cages. At 90 days of life, the male offspring from F1 were classified using the Lee index and established the F2 generation. Subsequently, the F2 generation rats, at 90 days of life, were mated with non-obese adults female rats (90 days of age), and their male offspring were also classified using the Lee index establishing the F3 generation.

The experimental groups were categorized as: Control group (CONT, n=14), MSG-induced obese rats (F1, n=14), Obese offspring (F2, n=14), and Grand offspring (F3, n=14). After assessing the Lee index, the animals from each generation had gastrointestinal motility, metabolic, and histological parameters evaluated and compared with the non-obese control group. All the variables were analyzed at the same age in all animals.

### Gastrointestinal motility

GI motility was evaluated by the alternating current biosusceptometry (ACB) technique. ACB uses non-invasive and radiation-free sensors to record variations on the biomagnetic field generated by an ingested magnetic material. The intensity of the signal is proportional to the amount of magnetic material and depends on the distance to the sensor (17). To measure the GI parameters (contractility and transit), all the animals fasted for 12 h. From then on, they were fed with a magnetic meal (1.6 g commercial chow plus 0.4 g ferrite powder) before the measurements. Ferrite (MgZnFe_2_O_3_, Imag, Brazil) is an inert ferromagnetic material that is not absorbed by the GI tract and is used as a magnetic marker. The ACB sensor was used to monitor the magnetic signals on the abdominal surface at 15 min intervals for at least 6 h (18).

For gastric contractility records, all the animals fasted for 12 h. Afterward, they ingested the magnetic meal described above and were anesthetized with 100 mg/kg ketamine (Cetamin®, Syntec, Brazil) plus 2.5 mg/kg acepromazine (Acepran®, Vetnil, Brazil), intraperitoneally. The animals were laid in a supine position and the ACB sensor was placed on the animal's abdominal surface in the corresponding anatomic region of the stomach. Magnetic signals were recorded continuously for 30 min at a sampling rate of 20 Hz, using a multichannel recorder (MP100 System; BIOPAC, USA) (17).

### Magnetic data analysis

To quantify the GI transit, the statistical moment was applied (19). The temporal average determined by magnetic intensity curves normalized by the area under the curve was employed to quantify the following parameters: mean gastric emptying time (MGET), which was defined as the time t (min) when a mean amount of magnetic meal was emptied from the stomach; mean cecum arrival time (MCAT), defined as the time t (min) when an increase occurred in the mean amount of magnetic meal that arrived in the cecum; and mean small intestinal transit time (MSITT), which was quantified by the difference between MCAT and MGET (18).

To quantify the gastric contractility parameters, all magnetic signals were analyzed in MatLab (Mathworks, Inc., USA) by visual inspection and thereafter using bi-directional Butterworth band-pass filters by Fast Fourier Transform (FFT). The highest frequency peak for each FFT was defined as the gastric dominant frequency and the smallest, as the signal noise. The amplitude of contraction (A) was calculated by the relationship between the power of gastric peak (P) and power of noise peak (P') and is expressed in decibels (dB) as follows: A = 10 log_10_ (P/P') (20).

### Serum and tissue sample collection

At the end of the GI motility evaluation, the animals were sacrificed at 120 days of life by anesthetic overdose through an intraperitoneal injection of ketamine (240 mg/kg, Cetamin®, Syntec) plus xylazine hydrochloride (45 mg/kg, Dopaser®, HertapeCalier, Brazil), followed by decapitation. Blood serum was sampled and stored under -80°C refrigeration until further assays.

### Histological preparations and measurements

Duodenum tissue samples were carefully collected and fixed with Metacarn (60% methanol, 30% chloroform, and 10% glacial acetic acid), dehydrated with serial alcohol, diaphanized in xylol, and embedded in paraffin. Histological cuts were performed with Micron HM 355S Automatic Microtomer (Thermo Scientific, Germany) with 4-μm thickness. The sections were stained with hematoxylin-eosin (H&E) and the images were captured on an optical microscope (Zeiss, Germany) coupled to a high-resolution camera (AxioCam ERc5s, Zeiss). Fifteen images per animal were randomly selected for morphometric analysis of the thickness of circular muscle and longitudinal muscle layers, villus height, and crypt depth using ImageJ software (NIH, USA). The villus height:crypt depth ratio was calculated (20).

### Glucose, insulin, and leptin serum quantifications

Glucose levels were measured immediately after serum collection by Glucose Liquiform enzymatic kit (Labtest, Brazil). ELISA immunoassays were used for leptin (Rat Leptin kit, Invitrogen, USA) and insulin (Rat Insulin kit, Elabscience Biotechnology Co. Ltd., USA) serum concentration analyses according to manufacturers' instructions.

### Statistical analysis

Kolmogorov-Smirnov test was employed to assess the normality of data. The data are reported as means±SD with a limit of statistical significance up to 5% (P<0.05). Analysis of variance (ANOVA) followed by Tukey's *post hoc* test was performed for the multiple comparisons.

## Results

Table 1 shows the frequency of obesity, Lee index, and body weight for all groups studied. From the MSG-induced obese rats (F1), 73.3% of offspring in the F2 generation, and 7.0% of grand offspring in the F3 generation were obese, according to the parameters evaluated. Considering these results, only obese animals from F1 and F2 generations, and non-obese animals from F3 generation continued in the study because they were the large majority in each group. Furthermore, body weight was significantly higher for F1 and F2 groups (obese generations) compared with CONT group, whereas body weight of F3 generation (non-obese) was not different from the CONT group.

**Table 1:** Frequency of obesity, Lee index, and body weight of Control (CONT), monosodium glutamate-induced obese rats (F1), obese offspring (F2), and grand offspring (F3) groups.

	CONT	F1	F2	F3
Obesity in the generations	0% (n=14)	100% (n=14)	77.8% (14/18)	7.0% (1/15)
Obesity in the experimental groups included in analysis	0% (n=14)	100% (n=14)	100% (n=14)	0% (n=14)
Lee index	<0.300	0.313±0.010	0.325±0.020	<0.300
Body weight (g)	296.1±23.1	341.8±20.7*	336.2±60.8*	329.8±26.4

Only the 14 obese animals from F2 and the 14 non-obese animals in F3 were included in the analysis. Data are reported as means±SD. *P<0.05 compared with CONT (ANOVA followed by Tukey's *post hoc*).

**Figure 1:**
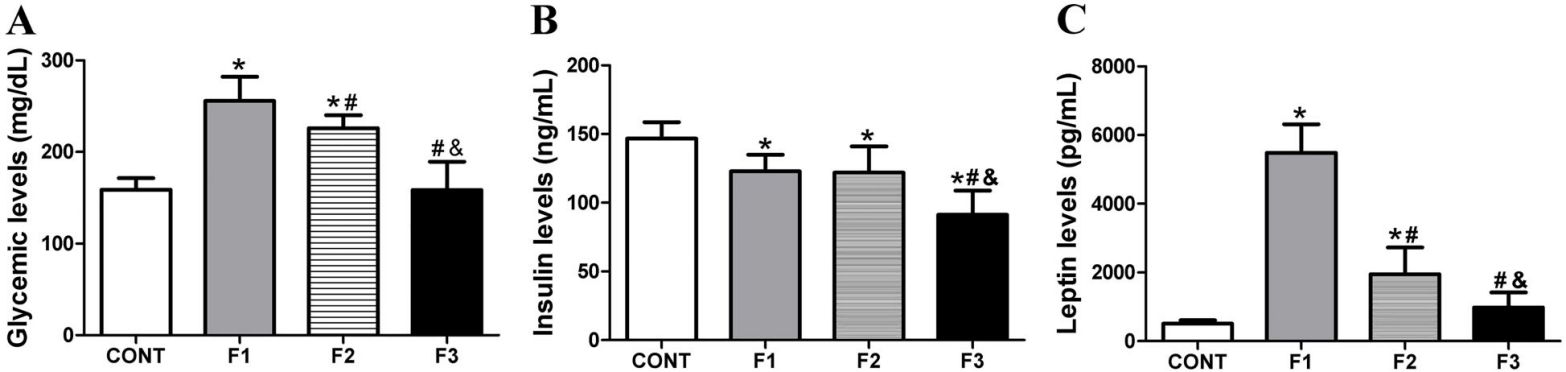
Glucose (**A**), insulin (**B**), and leptin (**C**) levels for Control group (CONT), monosodium glutamate (MSG)-induced obese rats (F1), obese offspring (F2), and grand offspring (F3). Data are reported as means±SD (n=14 per group). *P<0.05 compared with CONT, ^#^P<0.05 compared with F1, and ^&^P<0.05 compared with F2 (ANOVA followed by Tukey's *post hoc*).

Figure 1 shows the glucose, insulin, and leptin levels for F1, F2, and F3 generations. Glucose levels were higher for the obese groups, MSG-induced obese rats (F1) and F2 generation offspring compared with CONT and F3 (P<0.001). In addition, F2 showed a slightly lower glucose level compared with F1 (P<0.05). No differences were found between F3 generation and CONT groups. In F1 obese and F2 obese groups, insulin levels were lower compared to CONT (P<0.05). Insulin levels for F3 were significantly lower compared with CONT (P<0.01), F1 (P<0.05), and F2 (P<0.01) generations. Leptin levels were increased in the F1 and F2 obese groups compared to CONT (P<0.001 and P<0.01, respectively). The F1 group presented higher leptin levels than the F2 generation (P<0.001). Rats in the F3 generation had no difference in leptin levels compared to the CONT group.

**Figure 2:**
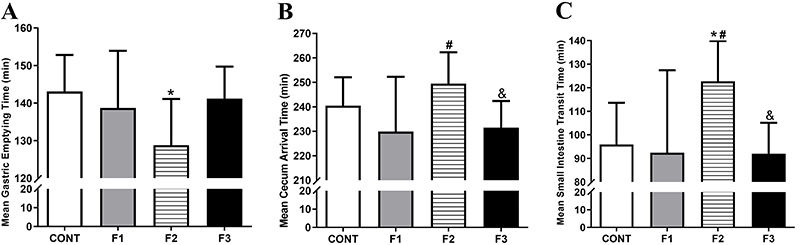
Gastrointestinal transit parameters evaluated for the Control group (CONT), monosodium glutamate (MSG)-induced obese rats (F1), obese offspring (F2), and grand offspring (F3). Mean gastric emptying time (MGET, **A**), mean cecum arrival time (**B**); mean small intestine transit time (**C**). Data are reported as means±SD (n=14 per group). *P<0.05 compared with CONT, ^#^P<0.05 compared with F1, and ^&^P<0.05 compared with F2 (ANOVA followed by Tukey's *post hoc*).

The GI transit parameters evaluated in all groups are shown in Figure 2. The F2 obese group had accelerated gastric emptying compared with the CONT group (P<0.05). No difference was found among F1, F2, and F3 groups for gastric emptying time. Cecum arrival in F2 was delayed compared with F1 (P<0.05) and F3 groups (P<0.05), although it was not different from the CONT group. The small intestine transit time in the F2 group was delayed compared with CONT, F1, and F3 groups (P<0.05).

**Figure 3:**
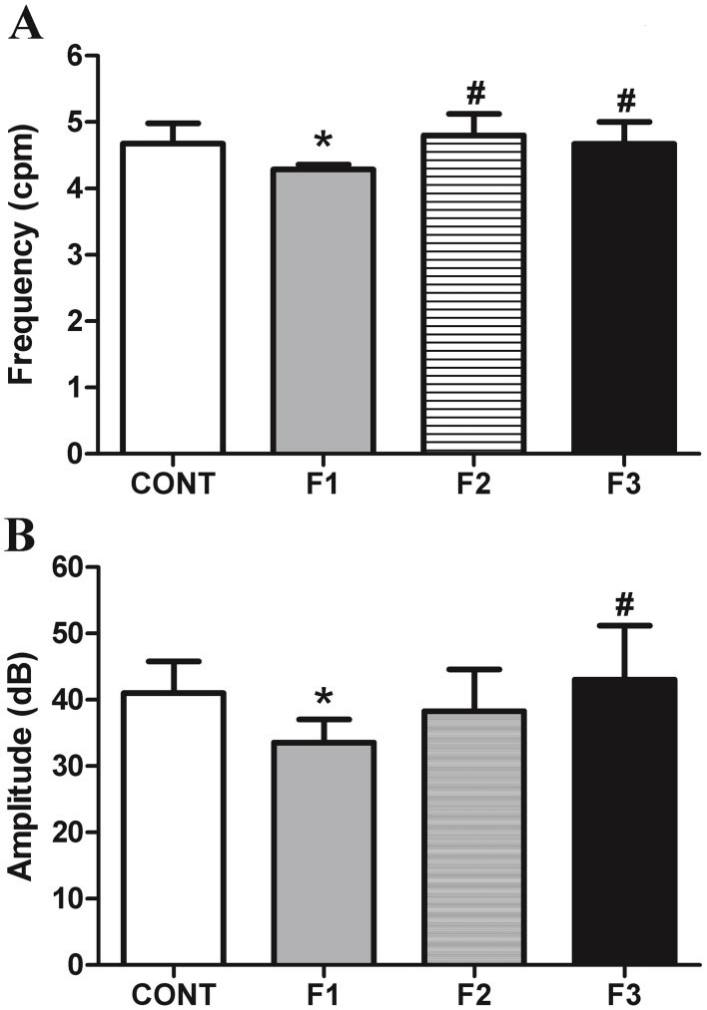
Gastric contractility parameters calculated for the Control group (CONT), monosodium glutamate (MSG)-induced obese rats (F1), obese offspring (F2), and grand offspring (F3). Frequency in contractions per min (cpm) (**A**) and amplitude (**B**) of gastric contractions. Data are reported as means±SD (n=14 per group). *P<0.05 compared with CONT, ^#^P<0.05 compared with F1 (ANOVA followed by Tukey's *post hoc*).

Gastric contractility parameters evaluated in all groups are shown in Figure 3. In the F1 group, the frequency of gastric contractions was reduced compared with CONT, F2, and F3 groups (P<0.01). No significant difference was found between the F2 and F3 generations compared to the CONT group. The amplitude of gastric contractions was similar between the CONT and F3 groups (41.30±4.78 dB and 43.10±8.10 dB), and both were significantly higher compared with the F1 generation (33.65±3.43 dB; P<0.05 and P<0.01, respectively).

**Figure 4:**
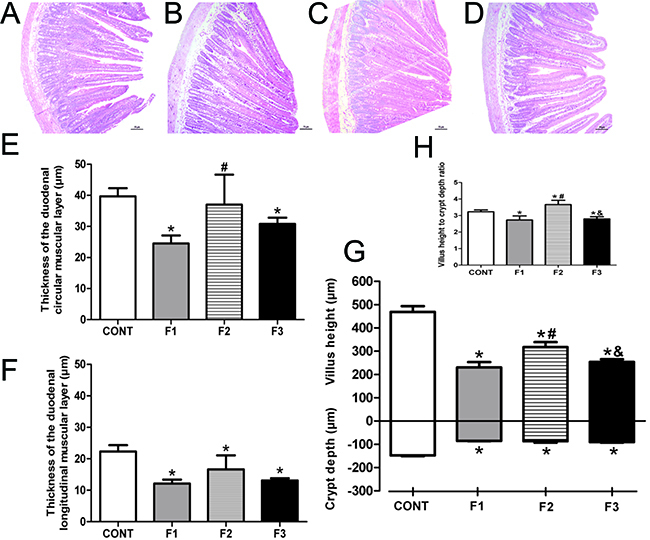
Histological photomicrographs of H&E staining of the duodenum (**A**-**D**) of Control (CONT), monosodium glutamate (MSG)-induced obese rats (F1), obese offspring (F2), and grand offspring (F3) (×10 objective, scale bar, 50 μm). Quantification of the thickness of the duodenal circular muscle layer (**E**) and longitudinal muscle layer (**F**), crypt depth, villus height (**G**), and villus height to crypt depth ratio (**H**). Data are reported as means±SD (n=14 per group). *P<0.05 compared with CONT, ^#^P<0.05 compared with F1, and ^&^P<0.05 compared with F2 (ANOVA followed by Tukey's *post hoc*).

Representative H&E-stained duodenum tissue and the histological and morphometric analyses are presented in Figure 4. The thickness of the circular muscle layer for the F1 and F3 groups was thinner compared with the CONT group (P<0.01), and in F1 it was significantly thinner than F2 (P<0.01). No significant difference was observed in the F2 group compared with the CONT group. In the longitudinal muscle layer, the thickness was even thinner in F1, F2, and F3 groups compared with CONT (P<0.01). All the generations presented both lower crypt depth and villus height compared with CONT (P<0.001). The F2 group presented greater villus height compared with the F1 and F3 groups (P<0.01). In this regard, villus height to crypt depth ratios in F1 and F3 groups were smaller compared with CONT (P<0.01). The F2 generation presented a higher villus height to crypt depth ratio compared with all groups (P<0.01 compared to CONT and P<0.001 compared to F1 and F3).

## Discussion

In this study, we demonstrated for the first time that MSG-induced obesity led to paternally derived, transgenerational obesity, and gastrointestinal alterations in male rats. The MSG-induced obese rats (F1) and their obese offspring generation (F2) showed disorders in GI motility with increased glucose and leptin levels. No functional changes or obesity phenotype was observed in the grand offspring generation (F3). All generations had changes in the muscular layers and duodenal mucosa, strengthening the extent of the disturbances driven by obesity.

Body weight was significantly higher for the F1 and F2 generations (obese rats) compared to the control group, whereas the F3 generations, characterized as non-obese, presented body weight that was not different from the control. The percentage of obesity was 100.0, 77.8, and 7.0% for the F1, F2, and F3 generations, respectively. This pattern indicated that MSG-induced obesity leads to paternally derived obesity in the subsequent generation (F2), with minor effects in the grand offspring generation (F3). Studies have shown that neonatal administration of MSG sets a precedent for the development of obesity due to the hypothalamic lesion resulting in a Lee Index with values higher than 0.300, typical physical characteristics, and delay in growth (12).

There are important interactions among adipose tissue, gut hormones, central nervous system, and GI tract in the development and maintenance of obesity (14,21). The investigation of the relationship between obesity and GI motility remains inconsistent and scarce, despite that it could represent an opportunity for novel therapeutic approaches for the treatment of obesity or other GI diseases (13,14).

Gastric emptying plays an important role in the intestinal exposure to nutrients and satiety (22,23), and it is closely related to glycemic control since it acts as a major determinant of postprandial glycemic regulation (24). In our study, both the F1 and F2 generations had changes in gastric motility and, in parallel, their glycemic levels were higher compared to the CONT and F3 groups. Our group has already demonstrated the relationship between these variables in mildly diabetic rats (25). The severity of gastric dysmotility and gastric repercussions seems to be inversely proportional to glycemic control (26).

Small bowel transit regulates nutrient absorption and seems to be related to the development of obesity (23). In our study, the F2 generation had a delayed MCAT and MSITT compared to all groups, which reinforced that some programmed effects can be transmitted to subsequent generations (27). As the absorption of nutrients from the small intestine is based on the efficiency of both digestive and epithelial transport mechanisms and the area of the intestinal mucosa, it is supposed that a delay in the intestinal transit may increase absorption and weight gain (15,23). Other studies have shown that small intestinal transit (13,21) and intestinal myoelectrical activity (13) were not affected by obesity induced by a high-fat diet (HFD).

Such conflicting findings regarding GI motility reported in obesity may be justified by different methodologies, analyses of the experimental data, and different species and/or strains used as animal models (13,28). Although several methods are available to evaluate GI motility, most of them are invasive or restricted by ethical issues (29). ACB is a magnetic technique validated to evaluate GI motor functions in rodents repeatedly (17,18,20). It is non-invasive and allows recording the GI motility physiologically in real time, without interfering with the system itself.

Despite motility changes, the gut is a target for histologic changes that need to be evaluated to handle the impact of the disease on different segments. In our study, the thickness of the longitudinal muscle layer in the duodenum was reduced in all generations; however, the circular muscle was reduced only in the F1 generation. Another study also observed a decrease of the small intestine wall thickness in MSG-induced obese rats, but subsequent generations were not evaluated (30).

Muscular layers offer different contributions to the intestinal motor patterns: the circular layer acts on the mixing motions of the luminal content, whereas the propulsion activity is triggered by the longitudinal layer (31). Based on these findings, we assume that thinner muscular layers could have slowed the intestinal transit in the F2 group. In addition, disorders on the myenteric plexus may also contribute to the damage on GI transit, since it is located between the longitudinal and circular layers of the muscular layer (32).

In duodenal mucosa, the height of the villi and the depth of the crypt as well as the villus:crypt ratio of the F1 and F3 generations were reduced. In the F2 group, there was a higher villus:crypt ratio compared with CONT, despite the height of the villi and depth of crypt being reduced. The small intestine absorptive function also depends on the villus distribution, and enterocyte and crypt integrity (33). Hence, while such a function may be compromised in F1 and F3, the greater villus height in F2 may have been an adaptive morphometric characteristic of this spontaneous obese generation to increase the intestinal absorptive area. The ileum of the MSG-induced obese mice had lower expression levels of the junction proteins increasing the intestinal permeability, suggesting that the damage of the intestinal barrier induces systemic inflammation (34).

Peptides secreted by adipocytes can modulate GI motility by acting on the central and enteric nervous system (14). Leptin is produced by adipose tissue and exerts its primary effect on the hypothalamic nuclei to regulate food intake and body weight (35). In addition, leptin seems to influence gastric emptying and small intestine motility (21,35). Obese rats from F1 and F2 generations had higher leptin levels compared to CONT and F3 groups. Specifically, in the F1 generation, leptin concentrations were higher compared to F2. There was no difference between leptin levels from F3 and CONT groups. Thus, we believe that this may be one of the factors involved in the changes in GI motility that was observed in obese generations.

In our study, a reduction in the serum insulin concentrations in the F1, F2, and F3 generations compared to CONT was observed. Insulin secretion dysfunction seems to be a more permanent change across generations (7) and could be a characteristic inherited from an obese father. In female offspring from a father submitted to the HFD with normal adiposity, β-cell alterations with impaired insulin secretion and glucose tolerance have been observed, showing the intergenerational transmission of the metabolic sequel from father to offspring (36).

It is important to investigate the paternal implications of the onset of obesity and other metabolic disorders in offspring. MSG has shown toxic effects on several systems (11,37), and offspring from MSG-induced obese rats can be used as a model for obesity studies since it impacts on offspring metabolism and body weight and can be compared to other models (6,37).

In humans, overweight parents are considered as a risk factor for offspring overweight/obesity (38). Children from obese parents have an increased risk for obesity, reaching 80% when both parents are obese, 40% when only one parent is obese, and 10% when none is obese (39). In experimental studies, 80% of offspring rats from obese females were also obese (6). In our study, the F2 generation also showed a similar obese percentage in males, corroborating the strong paternal role in the transmission of obesity as pointed out in human studies (40). Thus, our data showed that the father can onset the intergenerational transmission of obesity induced or inherited, and this condition can also affect the gastrointestinal function. Furthermore, our study can contribute to new approaches in the field of developmental programming by considering the influence of paternal obesity and its transgenerational effects on offspring health.

In conclusion, MSG-induced obese rats and their obese offspring generations had disorders in the gastrointestinal function associated with increased levels of glucose and leptin parallel to the reduction of insulin levels. From the third generation, GI variables were similar to control animals, despite morphometry being altered in all three generations. Our data added new insights about the physiology of obesity in rats and contributed to understanding the extent of the disturbances in gastrointestinal physiology provoked by a paternal cycle of obesity.
